# The coexistence of crowned dens sign and calcification of ligamentum flavum in the cervical spine: mere coincidence or meaningful association?

**DOI:** 10.1097/MD.0000000000042630

**Published:** 2025-06-06

**Authors:** Yake Meng, Lili Zhou, Rongxue Shao, Wei Zhang, Hao Pan, Yongfei Guo

**Affiliations:** a Department of Orthopedics, Hangzhou Hospital of Traditional Chinese Medicine, Zhejiang Chinese Traditional Medicine University, Zhejiang, China; b Department of Neurology, Hangzhou Hospital of Traditional Chinese Medicine, Zhejiang Chinese Traditional Medicine University, Zhejiang, China; c Department of Orthopedics, Shanghai Changzheng Hospital, The Naval Medical University, Shanghai, China.

**Keywords:** calcification, calcium pyrophosphate dihydrate, cervical ligamentum flavum, periodontoid calcification

## Abstract

We present a case series of cervical radiculomyelopathy caused by calcification of the ligamentum flavum (CLF), with or without concomitant periodontoid calcification. A brief literature review is presented, highlighting a potential relationship between these 2 conditions. In this retrospective study, we reviewed medical records of 33 patients diagnosed with cervical CLF. And we propose the term “crowned dens sign” (CDSign) to describe the characteristic radiographic finding of circumferential calcification around the odontoid process of the axis. Among the 33 patients, 28 (84.8%) were female and 5 (15.2%) were male, with a mean age of 70.6 years. Based on the cervical computed tomography images, a total of 81 cervical segments of CLF were recorded. The most commonly involved levels were C4-5 and C5-6, demonstrating the characteristic pattern of mid-cervical predominance. The CDsign was identified in 26 cases (79%), demonstrating a high prevalence in this cohort. The patient cohort comprised the following treatment groups: 2 patients received conservative management, twenty-three underwent posterior surgical procedures, and 8 were treated with anterior cervical surgery. The coexistence of CDSign and CLF represents an exceptionally rare clinical occurrence. The correlation between these 2 conditions extends beyond mere coincidence. The coexistence of CLF and CDSign may represent a rare but distinct cervical manifestation of calcium pyrophosphate dihydrate deposition disease.

## 1. Introduction

The ligamentum flavum, aptly named for its richness in yellow elastin, is a protective spinal ligament covering the posterior and lateral walls of the spinal canal. Degenerative changes of the ligamentum flavum is an important cause of spinal stenosis and spinal cord compression. The main pathological changes include hypertrophy, calcification, ossification, and cyst formation.^[1]^ The ossification of the ligamentum flavum (OLF) or calcification of the ligamentum flavum (CLF) predominantly occurs in the thoracic regions, while its occurrence in the cervical region is very infrequent.^[2]^ Histological investigations revealed that CLF may be a rare manifestation of the deposition of calcium pyrophosphate dihydrate (CPPD) crystals.^[3]^

Periodontoid calcification is an under-recognized entity, which is usually discovered incidentally during cervical spine computed tomography (CT) images. It has been reported that periodontoid calcification due to CPPD disease can result in acute neck pain.^[4]^ In this study, we report a case series of cervical radiculomyelopathy caused by CLF combined with or without periodontoid calcification/crowned dens sign (CDSign) radiologically. The probability of an association between these 2 conditions were discussed.

## 2. Materials and methods

This retrospective study obtained approval from the institutional review board of our esteemed hospital, Hangzhou Hospital of Traditional Chinese Medicine Ethics Committee. The program was conducted with the participants’ informed consent. A meticulous search of electronic medical records was carried out from September 2020 to January 2021, identifying the patients who had been diagnosed with OLF and/or CLF of the cervical spine. From these records, pertinent data related to the disease were summarized, included demographic characteristics (such as sex and age), clinical characteristics (chief complaint, and disease duration), radiological characteristics (such as lesion segment, contiguous/noninvolvement, concomitant periodontoid calcification, and associated degenerative changes), and treatment modalities. If available, the data of histological examination of calcified lesions were recorded. In our study, the calcification surrounding the dens was termed the CDSign. Throughout the study period, this comprehensive search yielded a total of 42 patients, of whom 9 cases were excluded due to incomplete imaging and poor image quality. Thus, the final number of subjects included in this study amounted to 33.

### 2.1. Statistical analyses

A statistical analysis was performed using *t* test and Fisher^’^s exact sign and a value of *P* < .05 was considered to be significant. All statistical analyses were conducted using the Statistical Package for the Social Sciences software program version 21.0 (SPSS, Chicago).

## 3. Results

A total of 33 cases were documented, which was 5 men and 28 women, and the fundamental clinical data were summarized in Table 1. Mean age was 70.6 years (56–86 years), and the average age of female patients was 69.9 years, while the male patients had an average age of 74.6 years (Table 1).

**Table 1:** Basic clinical data.

Case	Age/sex	Neck pain	Neurological symptoms	Duration	Morphology of CLF	Affected levels	Disc herniation	Concomitant CDSign	Treatment
1	69/F	Y	Radiculopathy	1 mo	Nodular	C4-7	Y	Y	A
2	79/F	NO	NO	0	Nodular	C3-6	N	Y	N
3	70/F	Y	Radiculopathy	3 mo	Nodular	C5-6	Y	Y	P
4	66/F	Y	Myelopathy	6 mo	Nodular	C2-6	Y	Y	P
5	54/F	Y	Myelopathy	8 d	Nodular	C4-6	N	Y	N
6	76/M	Y	Myelopathy	3 mo	Nodular	C4-7	Y	Y	P
7	70/F	Y	Radiculopathy	2 mo	Nodular	C4-7	Y	Y	P
8	70/F	Y	Myelopathy	3 mo	Nodular	C2-6	Y	Y	P
9	77/M	NO	Myelopathy	2 d	Nodular	C4-6	Y	Y	P
10	68/F	Y	Myelopathy	6 mo	Nodular	C3-7	Y	N	P
11	86/F	NO	Myelopathy	36 mo	Nodular	C4-5	N	N	P
12	82/F	NO	Myelopathy	24 mo	Nodular	C4-7	N	Y	P
13	76/M	NO	Myelopathy	10 mo	Nodular	C4-7	N	Y	P
14	78/M	NO	Myelopathy	7 d	Nodular	C4-T1	OPLL	Y	P
15	56/F	Y	Radiculopathy	6 mo	Nodular	C7-T1	Y	Y	A
16	58/F	Y	Radiculopathy	12 mo	Nodular	C6-7	Y	N	A
17	57/F	Y	Myelopathy	1 mo	Nodular	C4-6	N	Y	P
18	67/F	Y	Radiculopathy	10 mo	Nodular	C4-6	Y	Y	A
19	67/F	NO	Radiculopathy	12 mo	Nodular	C3-5	OPLL	Y	P
20	69/F	NO	Myelopathy	48 mo	Nodular	C4-7	N	Y	P
21	70/F	Y	Radiculopathy	5 mo	Nodular	C4-6	Y	Y	P
22	81/F	Y	Myelopathy	2 mo	Nodular	C3-T1	Y	Y	A
23	77/F	Y	Myelopathy	6 mo	Nodular	C5-6	N	Y	P
24	72/F	Y	Radiculopathy	2 mo	Nodular	C5-6	N	Y	P
25	66/M	Y	Myelopathy	6 mo	Nodular	C4-7	Y	Y	P
26	71/F	Y	Myelopathy	12 mo	Nodular	C4-5	Y	N	A
27	72/F	NO	Radiculopathy	50 mo	Nodular	C3-T1	Y	Y	A
28	73/F	Y	Radiculopathy	24 mo	Nodular	C4-6	N	Y	P
29	72/F	Y	Myelopathy	3 mo	Nodular	C3-5	Y	N	A
30	73/F	Y	Myelopathy	6 mo	Nodular	C5-7	Y	Y	P
31	75/F	NO	Myelopathy	4 mo	Nodular	C3-6	Y	Y	P
32	62/F	NO	Myelopathy	3 mo	Nodular	C4-5	Y	N	P
33	72/F	Y	Myelopathy	4 mo	Nodular	C3-5	Y	N	P

A = anterior surgery, CLF = calcification of the ligamentum flavum, d = days, F = female, M = male, mo = months, N = No, N = nonoperative treatment, OPLL = ossification of posterior longitudinal ligament, P = posterior surgery, Y = Yes.

### 3.1. Clinical characteristics and radiological assessment

Thirty-two patients presented with neurologic symptoms of radiculopathy and/or myelopathy, and 22 individuals suffered from intractable neck pain, whereas 1 patient did not report any complaints. The average duration of symptoms prior to diagnosis was 8.5 months, ranging from 2 days to 50 months (Table 1).

**Figure 1.**
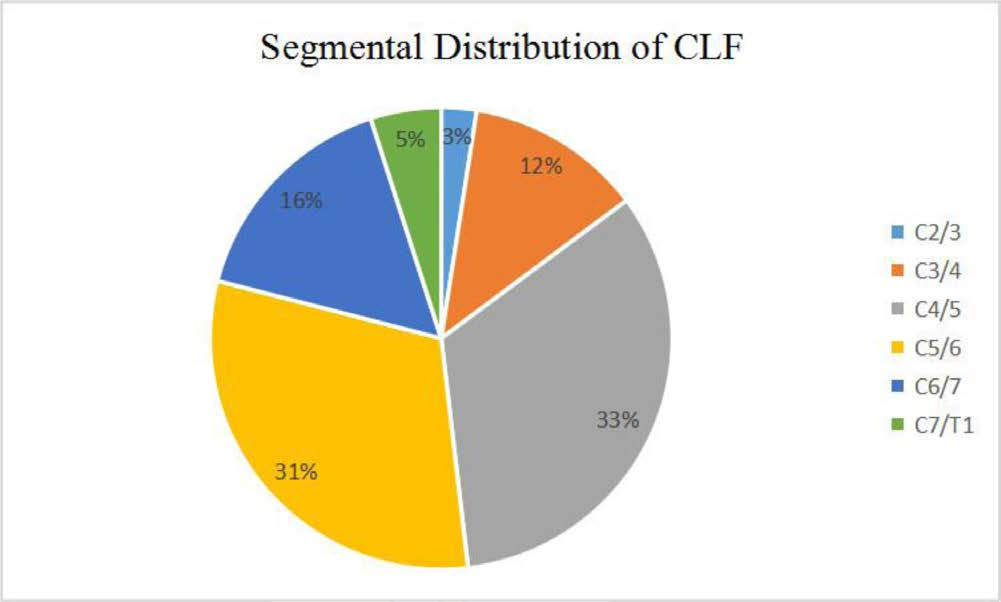
The segmental distribution of cervical ligamentum flavum calcification. CLF = calcification of the ligamentum flavum.

In all cases, cervical CT scans revealed the presence of oval or round-shaped calcifications positioned ventrally to the lamina. The precise locations of these calcifications were described, encompassing a total of 81 levels (Table 1). The affected segments included C2-3 in 2 cases, C3-4 in 10 cases, C4-5 in 27 cases, C5-6 in 25 cases, C6-7 in 13 cases, and C7-T1 in 4 cases. The calcification formation were single-segment in 8 cases (25%) and had multiple segments in 25 cases. Remarkably, the segment most frequently affected by calcification was C4-5, followed closely by C5-6 (Fig. 1). Furthermore, degenerative spondylolisthesis was observed in 21 patients, with a noteworthy tendency for spondylolisthesis to manifest at the same or adjacent segment as the margin of CLF. Additionally, 2 patients exhibited the presence of cervical ossification of the posterior longitudinal ligament, as indicated in Table 1.

### 3.2. Prevalence of the crowned dens sign

The CT images of 26 patients revealed the presence of curvilinear calcifications encircling the odontoid process of the axis, a phenomenon referred to as CDSign. This subset of 26 cases, characterized by the simultaneous occurrence of CDSign and CLF, was analyzed as a distinct group, exhibiting a prevalence rate of 79% (Table 1).

### 3.3. Treatment

**Figure 2.**
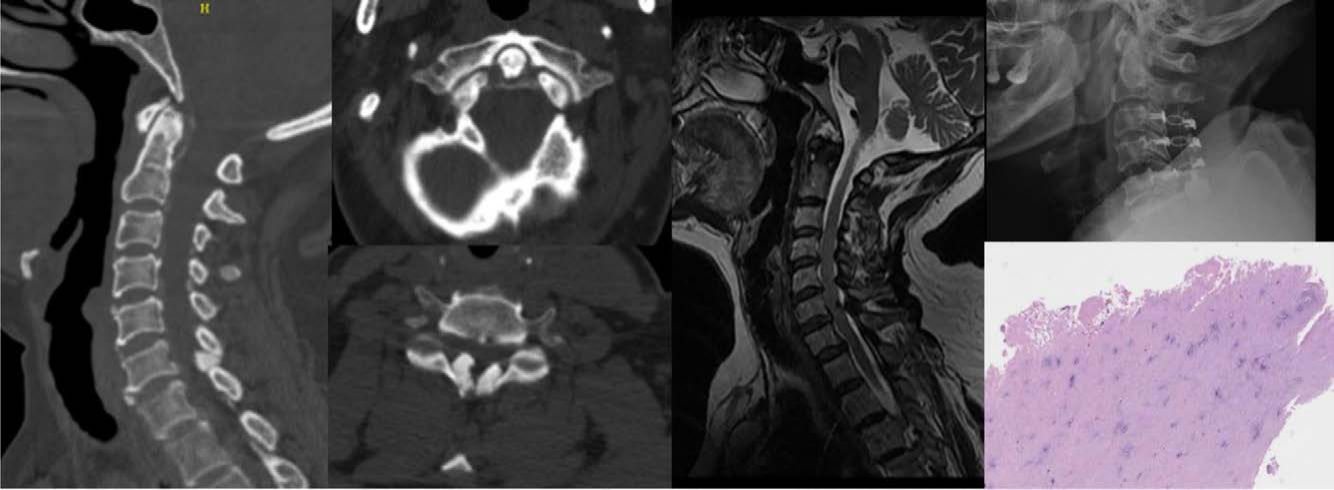
Cervical CT revealed calcification of the transverse ligament of atlas and multilevel ligamentum flavum calcification (predominantly at C6-7). Cervical MRI demonstrates cervical spinal canal stenosis with multilevel ligamentum flavum hypertrophy. Posterior cervical decompression postoperative X-ray examination. Histopathological examination of postoperative specimens revealed degenerated ligamentum flavum accompanied by dark blue calcifications. CT = computed tomography, MRI = magnetic resonance imaging.

**Figure 3.**
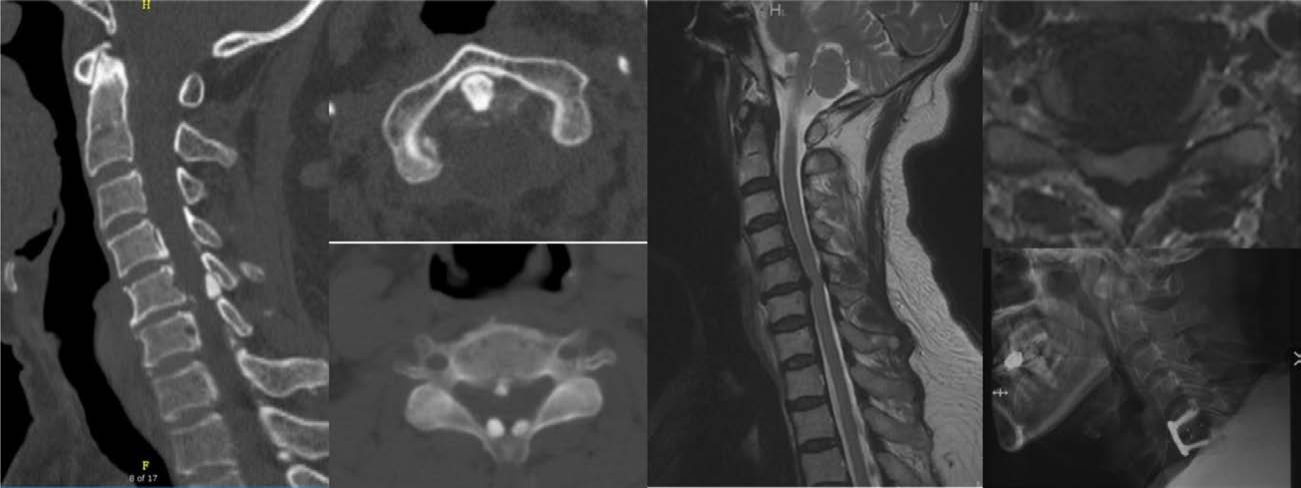
Cervical CT revealed calcification of the transverse ligament of atlas and ligamentum flavum calcification (predominantly at C5-6). Cervical MRI reveals C5/C6 disc herniation with ligamentum flavum hypertrophy causing spinal cord compression. The patient underwent C5/6 anterior cervical discectomy and fusion surgery. CT = computed tomography, MRI = magnetic resonance imaging.

Posterior cervical decompression surgery with instrumentation was performed on twenty-three patients (Fig. 2), while 8 patients underwent anterior cervical surgery (Fig. 3). Satisfactory outcome were obtained in all 31 patients. Histopathological examination of post specimens from 6 patients revealed degenerated ligamentum flavum accompanied by dark blue calcifications. With the exception of 2 patients, conservative treatment was administered (Table 1).

## 4. Discussion

OLF or CLF is an important cause of spinal stenosis that spinal surgeons often encounter.^[5]^ Liang et al^[5]^ found that the prevalence rate of OLF in the cervical spine were 0.25% based on 2000 patients with whole-spine CT images. In the clinical practice, there is often confusion between CLF and OLF due to insufficient histological examination. Although the 2 conditions share some similarities clinical presentation and treatment approaches, the pathological mechanisms are quite different.^[3]^ Calcification refers to the deposition of calcium or salt-related compounds in soft tissues, while ossification refers to the formation of mature trabecular bone. Radiologically, on a CT scan, CLF typically appears as hyperdense noncontiguous, oval-shaped mass, located at the posterolateral aspect of the lamina. In contrast, ossified lesions show irregular shape or V-shaped high-density masses along the lamina.^[6]^ CLF is a rare disease that typically occurs in the lower part of the cervical spine and predominantly affects females, whereas OLF frequently occurs in the upper or lower thoracic region.^[6]^ In our study, cervical CT scans were obtained in all patients, revealing oval-shaped, noncontiguous lesions adhered ventrally to the lamina. The pathological findings from 6 cases demonstrated degenerative ligamentum flavum with dark blue calcifications, consistent with those observed in CPPD diseases.

In 1976, Nanko et al^[7]^ reported the 1st documented case of symptomatic CLF in the cervical spine. In 1980, Kawano et al^[8]^ reported a case of cervical radiculomyelopathy caused by calcium pyrophosphate dihydrate crystal deposition in the ligamenta flava. Since then, there have been numerous reports of this condition. The pathophysiology of CLF remains largely unknown; however, previous studies have demonstrated that CLF is a relatively uncommon presentation of CPPD deposition disease.^[9]^ CPPD, also known as pyrophosphate arthropathy or pseudogout, is characterized by the deposition of CPPD crystals in articular and periarticular tissues. It primarily affects the peripheral joints, but can also affect the spine.^[10]^ In the spinal location, the cervical spine is the most commonly affected area, followed by the thoracic and lumbar regions. Advanced age is a significant risk factor for the development of CPPD crystal-associated arthritis, and CLF predominantly affects elderly women.^[3,6,10]^ Baba et al^[11]^ reported a series of 8 cases along with a comprehensive review of 91 reports on CLF. Among these cases, 85% were female, with an average age of 64.8 years. In our study, the male-to-female ratio was 1:6, and the mean age was 70.6 years, consistent with previous findings. It has been postulated that decreased estrogen levels at old age may be partially responsible for higher incidence of CLF in females.

Periodontoid calcification is a imaging findings defined by the radiographic calcifications in a crown-like configuration around the odontoid process.^[12]^The calcification is usually asymptomatic or causes acute neck pain and stiffness, which is defined as crowned dens syndrome (CDSyn).^[13]^ Sano et al^[14]^ reported the prevalence of CDSyn was 12.5% (11/88) in patients with periodontoid calcification. Based on a literature review and on our experience, CDSign were defined as calcifications around the odontoid process without clinical symptoms. In 2016, Kobayashi et al^[15]^ reported a case of patient with acute neck pain caused by calcified cervical yellow ligament with periodontoid calcification. Subsequently, Chang et al^[16]^ and Lee et al^[17]^ reported cases of cervical myelopathy caused by CLF with asymptomatic CDSign, respectively. The coexistence of CLF and CDSign is an exceedingly rare occurrence, only 3 similar cases have been reported in the English-language literature. It appears that the association between CDSign and CLF is either a rare entity or a mere coincidence. In our study, the incidence of CDSign were 79% (26 of 33) in patients with CLF. Our findings with more than half of the cases showing an overlap syndrome of CLF and concurrent CDSign, strongly suggest a significant association between these 2 conditions rather than a mere coincidence. A report by Lu et al.^[6]^ found that 72% (13/18) of patients with CLF had periodontoid calcifications. Muthukumar et al^[18]^ identified 2 types of CPPD crystal deposition in the cervical spine: calcifications of the ligamenta flava in the subaxial cervical spine (Type 1) and periodontoid calcifications in the upper cervical spine (Type 2). The exact relationship between these entities remains unknown, but the relatively frequent association among these rare diseases suggests that there may be a pathophysiological relationship among them and not only a simple coincidence. In our opinion, the combination of CLF and CDSign may represent a rare form of cervical manifestation of CPPD disease.

The patients with CLF of cervical spine exhibit a wide range of manifestations, from asymptomatic to radiculomyelopathy. Typical symptoms of CDSyn are acute neck pain, and neck stiffness. Within our study cohort, 22 patients reported experiencing axial neck pain, 11 patients presented with radicular symptoms, and 19 patients displayed signs of myelopathy. Notably, 26 patients exhibited the coexistence of CDSign, whereas its presence did not appear to significantly impact the clinical outcome of CLF treatment. Furthermore, a notable prevalence of cervical spondylosis was observed among the patients in our study with CLF. Posterior cervical spine surgery, a widely employed surgical technique for addressing various cervical spine disorders, has been extensively documented. Yang J et al^[19]^ reported 15 cases of cervical myelopathy caused by OLF, wherein all patients underwent bilateral laminectomy and achieved favorable clinical outcomes. In our study, 23 patients underwent posterior surgery, 8 patients received anterior surgery, and 2 patients were managed conservatively. The surgical interventions were successful, yielding satisfactory results. From our perspective, anterior decompression surgery is efficacious in treating patients with severe disc herniation and CLF.

### 4.1. Conclusions

CLF is an infrequent ailment. The simultaneous occurrence of CDSign and CLF is an atypical phenomenon. Our data has revealed a relatively elevated prevalence of concurrent CDSign in individuals with CLF of the cervical spine. The escalating number of reported cases exhibiting this dual affliction implies that the correlation between these 2 conditions surpasses mere happenstance. The coexistence of CLF with CDSign may represent a rare form of cervical manifestation of CPPD disease.

## Acknowledgments

I would like to express my sincere gratitude to all individuals and institutions that have contributed to the completion of this manuscript. Their support and encouragement have been invaluable to me.

## Author contributions

**Data curation:** Yake Meng.

**Formal analysis:** Yake Meng.

**Investigation:** Lili Zhou, Hao Pan.

**Methodology:** Lili Zhou, Hao Pan.

**Resources:** Rongxue Shao.

**Validation:** Wei Zhang, Yongfei Guo.

**Visualization:** Wei Zhang.

**Writing – review & editing:** Yongfei Guo.

**Writing – original draft:** Rongxue Shao.
